# High-risk HPV-associated ovarian squamous cell carcinoma: A case report and literature review

**DOI:** 10.1097/MD.0000000000035907

**Published:** 2023-11-03

**Authors:** Jianqi Li

**Affiliations:** Department of Obstetrics and Gynecology; Guangdong Provincial Key Laboratory of Major Obstetric Diseases; Guangdong Provincial Clinical Research Center for Obstetrics and Gynecology; Guangdong-Hong Kong-Macao Greater Bay Area Higher Education Joint Laboratory of Maternal-Fetal Medicine, The Third Affiliated Hospital of Guangzhou Medical University, Guangzhou, China.

**Keywords:** cervical neoplasia, high-risk HPV, mature cystic teratomas, ovarian cancer, ovarian squamous cell carcinoma

## Abstract

**Rationale:**

Ovarian squamous cell carcinoma (OSCC) is an exceedingly rare subtype, and high-risk human papillomavirus (HPV)-related OSCC is even rarer.

**Patient concerns:**

An 8-cm diameter ovarian cyst was detected during a routine B-ultrasound examination, and the patient underwent laparoscopic surgery. Postoperative pathological examination revealed HPV-16-related OSCC involving adjacent uterine tissue. The patient received postoperative radiotherapy and chemotherapy.

**Diagnoses:**

High-risk HPV-related OSCC.

**Interventions:**

No.

**Outcomes:**

The patient was finally diagnosed with high-risk HPV-related OSCC and underwent surgical treatment.

**Lessons subsections:**

In patients with high-grade cervical intraepithelial neoplasia who have undergone cervical conization and maintained annual HPV negativity, the possibility of high-risk HPV-related OSCC should be considered, despite its extreme rarity. Vigilance is essential in the presence of ovarian cysts even after HPV clearance.

## 1. Introduction

Currently, many diseases are known to be associated with high-risk human papillomavirus (HPV), including cervical intraepithelial neoplasia/cervical cancer, vulvar and vaginal intraepithelial neoplasia, and oral squamous cell carcinoma, among others. However, research on ovarian squamous cell carcinoma (OSCC) in relation to HPV remains scarce, and high-risk HPV-related OSCC is exceedingly rare, with only a few reported cases found in the literature. In this study, we present a case report of high-risk HPV-related OSCC and conduct a comprehensive review and analysis of the existing literature to provide valuable insights for clinical diagnosis and treatment.

## 2. Case report

**Figure 1.**
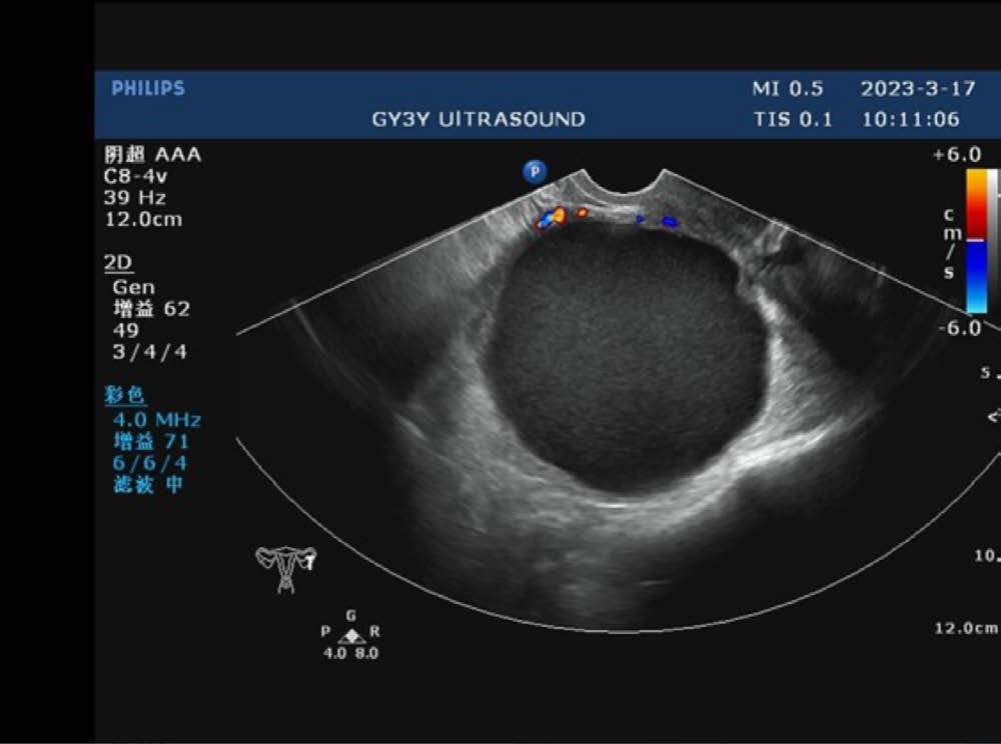
Routine gynecological ultrasound examination revealed a round-shaped hypoechoic area in the left ovary, measuring 81 × 74 mm, with clear borders and filled with fine dot-like low echoes.

**Figure 2.**
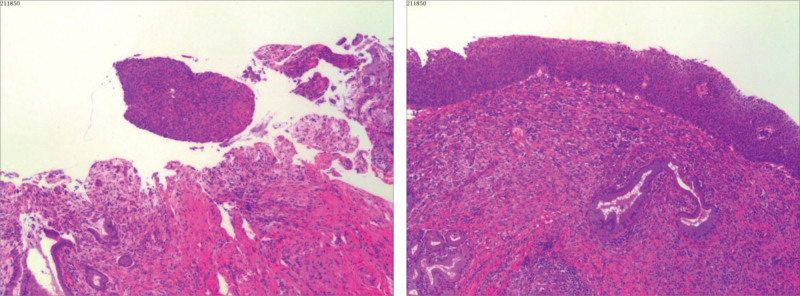
Cervical conization pathology in 2016: (Cervix 9 o’clock) High-grade cervical intraepithelial neoplasia (CIN-III) involving the glands; HPV DNA quantitative typing detected HPV-16 positivity. HPV = human papillomavirus.

**Figure 3.**
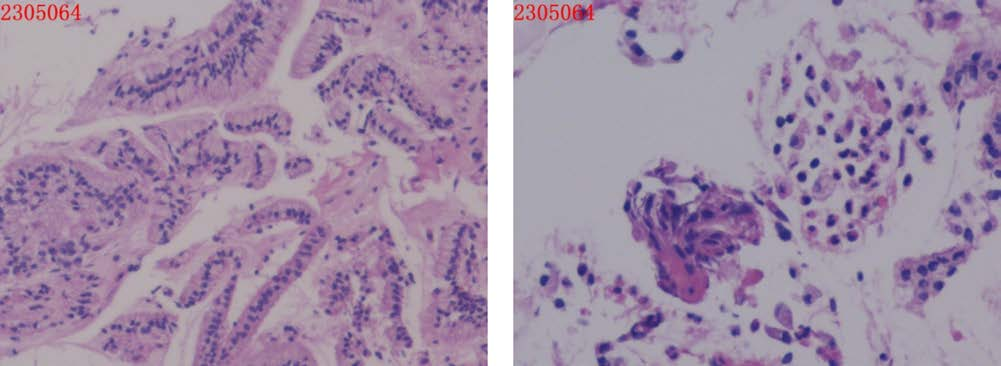
(ECC) Fragmented cervical canal mucosal epithelium and a small amount of squamous epithelium were observed, with no definite cancer cells. Immunohistochemistry: P16(-), Ki-67 (index 5%).

A 36-year-old female with HPV-16 positivity underwent colposcopy in 2016, leading to a diagnosis of high-grade cervical intraepithelial neoplasia (CIN-III) involving the glands. She underwent cervical conization, and subsequent annual follow-up examinations showed negative results for HPV and thinprep cytologic test (TCT). On March 17, 2023, during routine gynecological ultrasound examination, a round-shaped hypoechoic area measuring 81 × 74 mm with clear borders and filled with fine dot-like low echoes was found in the left ovary (Fig. 1). The patient also reported dysmenorrhea, raising the suspicion of ovarian endometriosis. Considering her history of HPV-16 positivity, previous CIN-III involving the glands, and cervical conization in 2016 (Fig. 2), preoperative cervical cancer screening was performed, showing negative results for HPV and TCT. As a precautionary measure, a vaginal examination was conducted along with cervical biopsy and endocervical curettage, both of which showed no abnormalities (Fig. 3).

**Figure 4.**
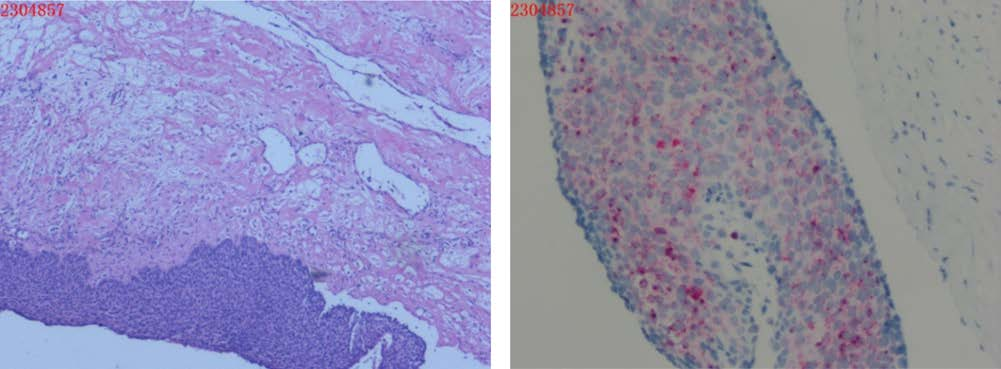
Postoperative pathology: a mass of grayish-white and grayish-red cyst wall tissue, measuring 805 and 616 mm, with a cyst wall thickness of 1 to 10 mm and a smooth outer surface. Additionally, a nodular tissue measuring 10 mm in diameter was observed, with blood clots attached to the inner wall. (Left ovarian cyst) The tumor exhibited predominantly solid-cystic features, mostly consistent with invasive squamous cell carcinoma of the HPV-related type. Some areas showed cystic changes with high-grade intraepithelial neoplasia. In the remaining left adnexa, ovarian endometriosis was observed with cyst formation. Molecular pathology analysis: Epstein-Barr virus detection (EBER−) was negative, while high-risk HPV RNA in situ hybridization (HPV+) was positive. HPV = human papillomavirus.

Subsequently, a laparoscopic exploration was performed, revealing an enlarged, cystic left ovary adhered to the left pelvic wall. The left fallopian tube, uterus, and right adnexa appeared normal, with no evidence of lesions or adhesions in the pelvic and abdominal organs or peritoneum. During the dissection of the left ovarian cyst, it ruptured, discharging brownish cystic fluid with no evidence of fatty tissue, hair, or teeth. Nodule-like tissues were observed at the adhesion sites, measuring approximately 1.5 × 1.0 cm. The postoperative pathology indicated that the tumor in the left ovarian cyst was predominantly invasive squamous cell carcinoma, with a high-risk HPV association. Some areas exhibited cystic changes with high-grade intraepithelial neoplasia. The remaining left adnexa showed endometriosis with cyst formation. Molecular pathology analysis showed negative results for Epstein-Barr virus detection (EBER−) and positive results for high-risk HPV RNA in situ hybridization (Fig. 4).

**Figure 5.**
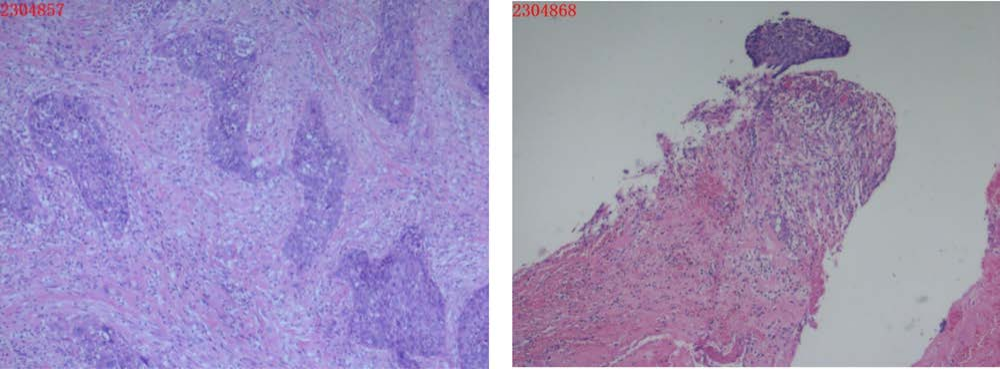
(Uterus + right adnexa) Pathology: Squamous cell carcinoma infiltration was observed from the outer layer of the cervix to the parametrial tissue, suggesting a correlation with the ovarian squamous cell carcinoma infiltration.

Subsequently, the patient underwent total hysterectomy, bilateral adnexectomy, pelvic lymph node dissection, omentectomy, and lysis of abdominal adhesions under general anesthesia. Pathological examination of the uterus and right adnexa revealed no CIN or squamous cell carcinoma components. However, squamous cell carcinoma infiltration was observed from the outer layer of the cervix to the parametrial tissue, indicating a correlation with the OSCC (Fig. 5).

## 3. Literature review and analysis

In 1974, German virologist Harold zur Hausen first discovered HPV and confirmed its close association with the development of cervical cancer.^[1]^ In 2005, the International Agency for Research on Cancer highlighted that persistent HPV infection is a direct factor leading to cervical cancer and its precancerous lesions.^[2]^

OSCC is a rare and invasive gynecological cancer. While most cases arise from mature teratomas, primary ovarian squamous cell carcinoma (POSCC) is believed to be linked to HPV infection. Diagnosing OSCC is challenging, requiring the exclusion of metastases from bladder, reproductive tract, lung, and gastrointestinal cancers.^[3]^ Malignant transformation in ovarian teratomas is a rare but well-documented phenomenon, with squamous cell carcinoma accounting for 80% of cases. In mature cystic teratomas, squamous cell carcinoma is mostly observed in women over 50 years of age, with elevated levels of squamous cell carcinoma antigen and cancer antigen CA125, and tumor size exceeding 100 mm.^[4,5]^

To date, there have been very few reports on high-risk HPV-related OSCC, and they are all individual case studies. Mai et al^[6]^ proposed a possible causal relationship between HPV and POSCC in middle-aged patients with high-grade cervical lesions. Pins et al^[7]^ reported a case of a 55-year-old patient with OSCC, where CIN was found to extend along the upper reproductive tract epithelium and ovarian surface, and HPV DNA was detected through polymerase chain reaction in the cervix, endometrium, fallopian tubes, and ovarian tumors, indicating the involvement of HPV in this case. Manolitsas et al^[8]^ presented a typical case of synchronous squamous intraepithelial lesions in the ovaries and cervix. Verguts ^[9]^ reported a case of a 48-year-old female who underwent cervical conization for CIN lesions in December 1997. Subsequently, 15 years later, she was diagnosed with ovarian squamous cell carcinoma with bone metastasis and received radiotherapy and chemotherapy, finally confirmed to be related to HR-HPV. In a study exploring a total of 15 cases of POSCC, Xi et al^[10]^ found that 5 cases (5/15) were positive for p16 expression and confirmed high-risk HPV infection by in situ hybridization, and these patients had longer overall survival time (*P* = .038) and progression-free survival time (*P* = .045) compared to HPV-negative patients. However, it should be noted that Shi et al^[11]^ suggested HPV-16 infection could induce OSCC in mature cystic teratomas, but upon review, the authors only detected HPV-16 infection in the OSCC tissue, which does not necessarily imply that HPV-16 caused the OSCC in the mature cystic teratoma.

The case presented in this study was diagnosed with HPV-16-related OSCC during laparoscopic exploration for an ovarian cyst detected by B-ultrasound. A review of the patient’s medical history revealed HPV-16 positivity in 2016, CIN-III involving the glands, and cervical conization with negative margins. Subsequent annual follow-ups showed negative results for HPV and TCT. Although preoperative vaginal examination and cervical scraping excluded cervical lesions or cervical cancer, the diagnosis of OSCC involving adjacent uterine tissue was confirmed, and HPV-16 positivity was detected in the tissue, while no cervical lesions were found.

In conclusion, based on the literature analysis, the authors consider the possibility that HPV-16 may have persisted in the upper reproductive tract for many years and eventually induced squamous cell carcinoma in the ovary, without causing the more common high-risk HPV-related cervical or vaginal cancers. This case is exceptionally rare and offers valuable clinical insights. For patients with CIN-III who have undergone cervical conization with subsequent annual HPV-negative follow-ups, the possibility of high-risk HPV-related OSCC should be considered, even though it is extremely rare.

## Author contributions

**Conceptualization:** Jianqi Li.

**Data curation:** Jianqi Li.

**Formal analysis:** Jianqi Li.

**Investigation:** Jianqi Li.

**Methodology:** Jianqi Li.

**Software:** Jianqi Li.

**Supervision:** Jianqi Li.

**Validation:** Jianqi Li.

**Visualization:** Jianqi Li.

**Writing—original draft:** Jianqi Li.

**Writing—review & editing:** Jianqi Li.
